# A Study on How Industrial Pharmacists Rank Competences for Pharmacy Practice: A Case for Industrial Pharmacy Specialization

**DOI:** 10.3390/pharmacy4010013

**Published:** 2016-02-06

**Authors:** Jeffrey Atkinson, Kristien De Paepe, Antonio Sánchez Pozo, Dimitrios Rekkas, Daisy Volmer, Jouni Hirvonen, Borut Bozic, Agnieska Skowron, Constantin Mircioiu, Annie Marcincal, Andries Koster, Keith Wilson, Chris van Schravendijk

**Affiliations:** 1 Pharmacolor Consultants Nancy, 12 rue de Versigny, Villers 54600, France; 2 Pharmacy Faculty, Vrije Universiteit Brussel, Laarbeeklaan 103, Brussels 1090, Belgium; kdepaepe@vub.ac.be; 3 Faculty of Pharmacy, University of Granada (UGR), Campus Universitario de la Cartuja s/n, Granada 18701, Spain; sanchezp@ugr.es; 4 School of Pharmacy, National and Kapodistrian University Athens, Panepistimiou 30, Athens 10679, Greece; rekkas@pharm.uoa.gr; 5 Pharmacy Faculty, University of Tartu, Nooruse 1, Tartu 50411, Estonia; daisy.volmer@ut.ee; 6 Pharmacy Faculty, University of Helsinki, Yliopistonkatu 4, P.O. Box 33-4, Helsinki 00014, Finland; jouni.hirvonen@helsinki.fi; 7 Faculty of Pharmacy, University of Ljubljana, Askerceva cesta 7, Ljubljana 1000, Slovenia; Borut.Bozic@ffa.uni-lj.si; 8 Pharmacy Faculty, Jagiellonian University, UL, Golebia 24, Krakow 31-007, Poland; askowron@cm-uj.krakow.pl; 9 Pharmacy Faculty, University of Medicine and Pharmacy “Carol Davila” Bucharest, Dionisie Lupu 37, Bucharest 020021, Romania; constantin.mircioiu@yahoo.com; 10 European Association of Faculties of Pharmacy, Faculty of Pharmacy, Université de Lille 2 , Lille 59000, France; annie.marcincal@pharma.univ-lille2.fr; 11 European Association of Faculties of Pharmacy, Department of Pharmaceutical Sciences, Utrecht University, PO Box 80082, Utrecht 3508 TB, The Netherlands; A.S.Koster@uu.nl; 12 Applied Health Research Unit, School of Life and Health Sciences, Aston University, Birmingham B4 7ET, UK; k.a.wilson@aston.ac.uk; 13 Medical Faculty, Vrije Universiteit Brussel, Laarbeeklaan 103, Brussels 1090, Belgium; chrisvs@vub.ac.be

**Keywords:** education, specialization, practice

## Abstract

This paper looks at the way in which industrial pharmacists rank the fundamental competences for pharmacy practice. European industrial pharmacists (*n* = 135) ranked 68 competences for practice, arranged into 13 clusters of two types (personal and patient care). Results show that, compared to community pharmacists (*n* = 258), industrial pharmacists rank competences centering on research, development and production of drugs higher, and those centering on patient care lower. Competences centering on values, communication skills, *etc.* were ranked similarly by the two groups of pharmacists. These results are discussed in the light of the existence or not of an “industrial pharmacy” specialization.

## 1. Introduction

Graduates with a pharmacy degree are employed in a variety of positions, the most important (in terms of numbers) being community, hospital and industrial pharmacy. The discussion on whether these three domains require specific skills with a specific education has long been contentious. Industrial pharmacy as a university discipline is recognized by some European pharmacy departments. The PHARMINE study (*Pharmacy Education in Europe*) reported that pharmacy departments in 10/31 European countries give elective pre-graduate courses in industrial pharmacy, and 11/31 departments give post-graduate industrial pharmacy courses [1]. Most of the graduates from such courses go on to to work in an industrial setting. The PHARMINE study reported that a substantial number (37,308) of European pharmacists (6% of the industrial workforce) work in the pharmaceutical industry [2]; this is similar to the worldwide figure of 10% given by the International Pharmaceutical Federation [3].

In some European countries the status of the industrial pharmacist is officially recognized. In France, the profession of “industrial pharmacist” is defined by national law and the statutes of the pharmacy professional body [4]. On the global European level, this is not the case. In the European Union (EU), the 1985 EU directive on the profession of pharmacy [5], and the 2013 update [6], do not recognize any specialization in pharmacy (although these are recognized in medicine).

There is a second EU directive that is relevant in this case, however, and that is the EU directive on qualified persons working in the pharmaceutical industry [7]. In some EU member states, such as Germany [8], only those with a pharmacy degree meet the requirements set down in the qualified persons directive.

It appears, therefore, that the argument is equivocal for the existence of a specialized pharmacy job description of “industrial pharmacist” (*i.e.*, a pharmacist working in industry) that is different from that of other specialties such as community pharmacy. This paper looks at one aspect of this discussion: whether industrial pharmacists rank competences for practice differently than do community pharmacists. It is possible that pharmacists working within such specialties view the pharmacy profession differently. Within this context we investigated, therefore, whether the ranking by industrial pharmacists of competences for practice is different from that of community pharmacists.

In the PHAR-QA (“*Quality Assurance in European PHARmacy Education and Training*”) project [9], we asked community and industrial pharmacists to rank competences for pharmacy practice. This paper describes the similarities and differences between the ways in which European industrial and community pharmacists respectively rank competences for pharmacy practice.

## 2. Experimental Section

Ranking data on competences for practice were obtained using the PHAR-QA *surveymonkey* [10] questionnaire that was available online from 14 February 2014 to 1 November 2014, *i.e.*, 8.5 months [11]. Respondents came from 36/49 countries of the European Higher Education Area [12].

The first six questions were on the profile of the respondent (duration of practice, country of residence, current occupation (industrial, community… pharmacist)). There was also a question on the job title. This allowed a subdivision of industrial pharmacists according to their experience/activity: regulatory affairs, research and development, *etc.* A similar subdivision of the activities of community pharmacists was not possible, as all respondents in this group were involved in some form of dispensation to patients.

Questions 7 through 19 asked about 13 clusters of 68 competences (see Appendix A). Questions in clusters 7 to 11 were concerned with personal competences, and in clusters 12 to 19 with patient care competences.

Respondents were asked to rank the proposals for competences on a 4-point Likert scale:

(1) Not important = Can be ignored;
(2) Quite important =Valuable but not obligatory;
(3) Very important = Obligatory, with exceptions depending upon field of pharmacy practice;
(4) Essential = Obligatory.

There was also a “cannot rank” possibility as well as that of leaving the answer blank.

Results are presented in the form of “scores” calculated as follows:

Ranking score = (frequency rank 3 + frequency rank 4) as % of total frequency, which represents the percentage of respondents that considered a given competence as “obligatory”.

This calculation is based on a similar calculation made by the MEDINE consortium that studied the ranking of competences for medical practice [13]. Such scores are used for descriptive purposes only, and no conclusions on statistical differences amongst groups are based on scores.

Leik ordinal consensus [14] was calculated as an indication of the dispersion of the data using an Excel spreadsheet. The original Leik paper cited previously gives an explicit mathematical example of the calculation of ordinal consensus. Responses for consensus were arbitrarily classified as: < 0.2 poor, 0.21–0.4 fair, 0.41–0.6 moderate, 0.61–0.8 substantial, > 0.81 good, according to the scale used in MEDINE study.

The statistical significance of differences between rankings of competences or between rankings by different categories of respondents was tested by the chi-square test (confidence level 95%). Statistical tests were performed using GraphPad software [15].

Results are presented at 2 levels: that of the 13 clusters and that of the 68 competences.

Respondents could also add their comments on the different clusters. An attempt was made to analyze comments using the NVivo10 program [16] for the semi-quantitative analysis of unstructured data. It was found that the numbers involved were too small to draw significant conclusions.

## 3. Results and Discussion

The distribution by duration of practice of the groups is given in Table 1.

**Table 1:** Distribution of duration of practice (years) in industrial and community pharmacist responders. *n*: number in each category.

Respondents	< 5	6–10	11–20	21–40	Blank	Total
Industrial pharmacists (*n*)	26	31	28	23	27	135
Community pharmacists (*n*)	50	51	41	56	60	258

Most respondents had less than 20 years of experience, thus in both cases a relatively “young” population was involved. This may be due to the higher motivation of a younger population to reply to a questionnaire.

Respondents came from 36 European countries. Nineteen countries provided 5 or more respondents to one or both groups; they were: Belgium, Croatia, Czech Republic, Estonia, Finland, Germany, Greece, Hungary, Ireland, Italy, Macedonia, Montenegro, Norway, Romania, Slovenia, Spain, Switzerland, The Netherlands, and the United Kingdom.

The numbers of industrial pharmacy respondents arranged according to experience/activity are given in Table 2.

**Table 2:** Numbers of industrial pharmacy respondents subdivided according to experience/activity.

Experience/Activity	Number
Management	24
Regulatory affairs	23
Research and development	18
Quality assurance/compliance	16
Pharmaceutical technology	10
Clinical/medical affairs	8
Pharmacovigilance	5
Qualified person	5
Marketing and sales	5
Research student/Ph.D.	1
“Industrial pharmacist” or “pharmacist”	8
Blank	12

Table 2 shows that, while some respondents work in a typically “pharmaceutical” environment (such as regulatory affairs and pharmaceutical technology), many others work in more “generic” environments (such as management and quality assurance). If we consider that industrial surroundings correspond to 4 groups/stages, namely (1) research and development; (2) production; (3) analyses and quality assurance; and (4) marketing and sales, it appears that not all pharmacists employed in marketing and sales feel that they are industrial pharmacists. Indeed, marketing and sales representatives of “big pharma” are sometimes very far from classical industrial surroundings, even though they are employed in industry.

Table 3 shows the overall distribution of rankings by industrial and community pharmacists.

**Table 3:** Overall distribution (over 13 clusters of 68 competences) of rankings by industrial and community pharmacists.

Ranking	Industrial Pharmacists		Community Pharmacists	
Number of respondents	138		258	
Theoretical number of replies	9384 (= 138 × 68)		17,544 (= 258 × 68)	
Rank	Number	%	Number	%
4	2510	26.8	6643	37.9
3	3502	37.3	6002	34.2
2	1876	20.0	3076	17.5
1	432	4.6	608	3.5
Cannot rank + blanks	1064	11.3	1215	6.9
Score (%)	=((2510 + 3502)/8320) × 100) = 72.3		= ((6643 + 6002)/16,329) × 100 = 77.4	
Leik ordinal consensus	0.58		0.55	

Notes: Chi-square test on distribution of ranks for industrial *versus* community pharmacists: *p* < 0.05 (degrees of freedom = 3, ((4 ranks −1) × (2 groups −1)).

All but 7–11% of respondents were able to rank all competences. This suggests that the majority in both groups of respondents believed that they were sufficiently informed to reply to almost all the questions asked.

As judged from the Leik ordinal consensus values, dispersion was low. This suggests that both groups were relatively homogeneous and that subgroups with responses significantly different from the overall group do not exist. Similar values for ordinal consensus have been reported by the MEDINE consortium.

Overall ranking by industrial pharmacists was significantly lower (72%) than that by community pharmacists (77%). This raises the question of whether industrial pharmacists globally believed that the competence framework was less applicable; however, the global score was high with almost 3/4 of industrial pharmacists considering the competences “obligatory”. The global lower score of industrial pharmacists was weighted by the low scores they gave to patient care competences (see later).

**Figure 1:**
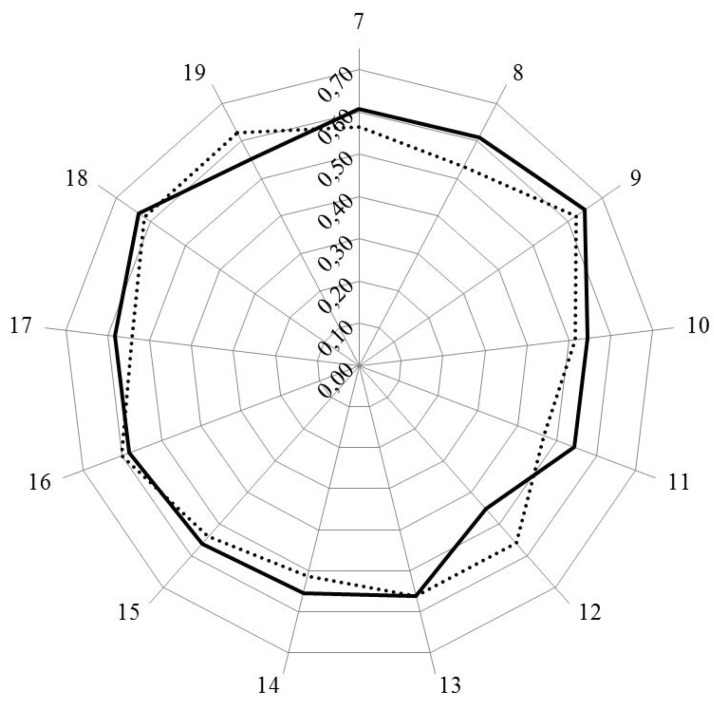
Leik ordinal consensus for rankings by clusters for industrial (solid line) and community (dotted line) pharmacists.

**Figure 2:**
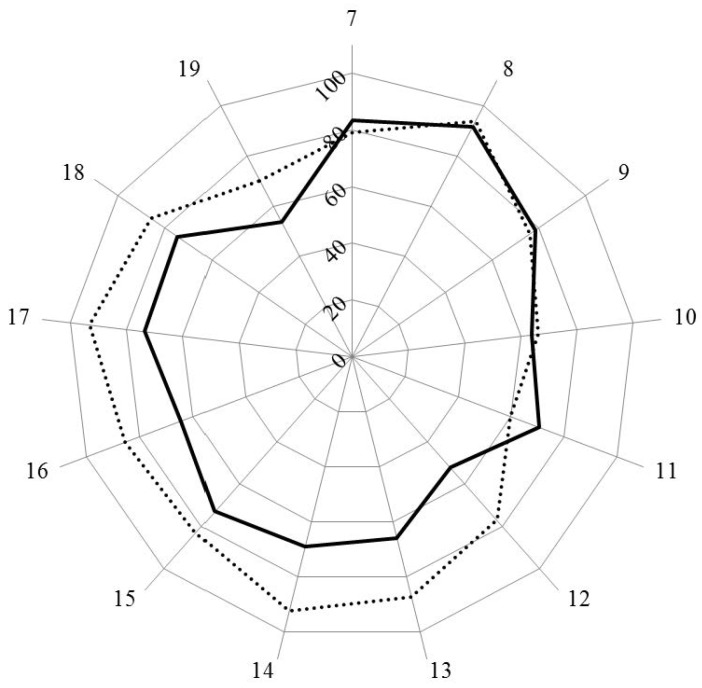
Scores (%) for rankings by clusters for industrial (solid line) and community (dotted line) pharmacists.

Figure 1 and Figure 2 show the results for analysis by clusters. In Figure 1, the values for Leik ordinal consensus are shown.

Leik ordinal consensus was higher for industrial pharmacists in 10/13 clusters including cluster 11 that dealt with competences for industrial pharmacy.

Scores for personal competences (clusters 7 to 11) were similar in industrial and community pharmacists, except for cluster 11, dealing with industrial pharmacy, for which industrial pharmacists scored higher. Scores for clusters dealing with patient care competences (clusters 12 to 19) were lower for industrial pharmacists.

**Figure 3:**
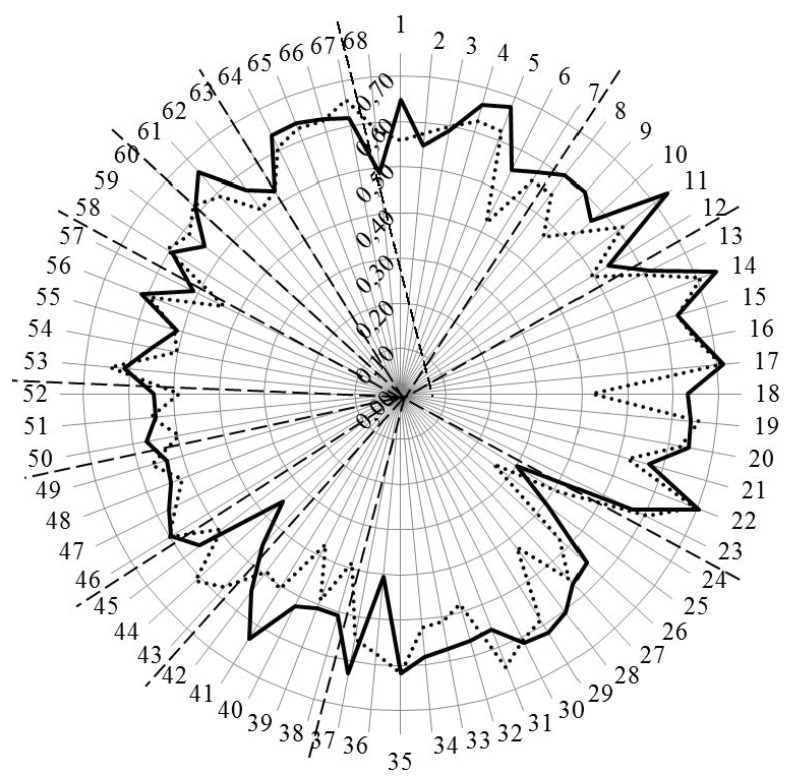
Leik ordinal consensus for rankings by competences for industrial (solid line) and community (dotted line) pharmacists. Dashed lines separate the different clusters of competences.

**Figure 4:**
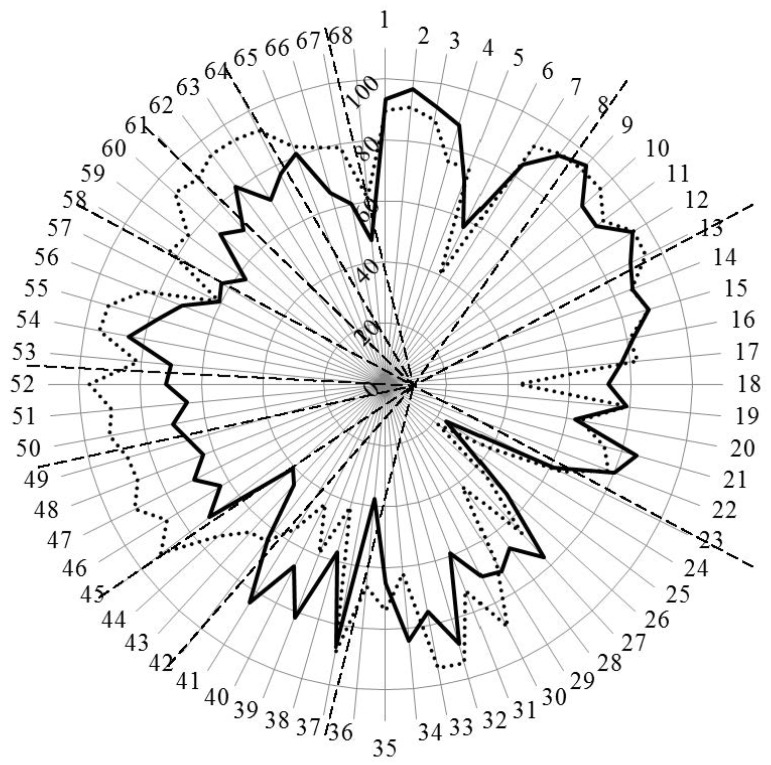
Scores (%) for rankings by competences for industrial (solid line) and community (dotted line) pharmacists. Dashed lines separate the different clusters of competences.

Figure 3 and Figure 4 show the results for analysis by competences. In Figure 3, the values for Leik ordinal consensus are shown.

Overall, the ordinal consensus values were higher for industrial than for community pharmacists. This was especially true for competences 6 (research), 18 (development, production of medicines), 28 (analytical chemistry), 38 (design, synthesis, *etc.* of active substances), 40 (EU directive on qualified persons), and 41 (drug registration, licensing and marketing). For all these competences, the consensus for industrial pharmacists was higher than community pharmacists (Figure 4 and Appendix A). For competences 31 (microbiology) and 44 (diagnostic tests) the ordinal consensus for community pharmacists was higher than that for industrial pharmacists, as were the scores (Figure 4 and Appendix A).

Scores for the 68 competences are given in Figure 4 and the Appendix A.

This graph shows that competences on the right-hand side concerned with personal values, subject matters and industrial pharmacy were often ranked higher by industrial pharmacists. Competences on the left-hand side concerned with patient care were often ranked higher by community pharmacists. A proviso must be added here; through a comparison between Figure 4 with Figure 3, it is obvious that, for competences scoring low (e.g., 24 and 25), consensus was low. Thus, the low ranking was far from unanimous.

Going into more detail, Table 4 shows the competences for which industrial pharmacist ranked higher (upper) and for which community pharmacists ranked higher (lower).

Table 4Competences ranked higher by industrial pharmacist (upper) and by community pharmacists (lower). (**a**) Industrial > community; (**b**) Community > industrial.

**Table:** (**a**)

n	Competence
4	Capacity to evaluate scientific data in line with current scientific and technological knowledge
6	Ability to design and conduct research using appropriate methodology
18	Ability to design and manage the development processes in the production of medicines
25	Physics
28	Analytical chemistry
34	Pharmaceutical technology including analyses of medicinal products
38	Current knowledge of design, synthesis, isolation, characterization and biological evaluation of active substances
39	Current knowledge of good manufacturing practice (GMP) and of good laboratory practice (GLP)
40	Current knowledge of European directives on qualified persons (QPs)
41	Current knowledge of drug registration, licensing and marketing

**Table:** (**b**)

n	Competence
24	Plant and animal biology
30	Anatomy and physiology; medical terminology
33	Pharmacotherapy and pharmaco-epidemiology
36	Pharmacognosy
43	Ability to perform and interpret medical laboratory tests
44	Ability to perform appropriate diagnostic or physiological tests to inform clinical decision making (e.g., measurement of blood pressure)
45	Ability to recognise when referral to another member of the healthcare team is needed because a potential clinical problem is identified (pharmaceutical, medical, psychological or social)
46	Retrieval and interpretation of relevant information on the patient’s clinical background
47	Retrieval and interpretation of an accurate and comprehensive drug history if and when required
48	Identification of non-adherence and implementation of appropriate patient intervention
49	Ability to advise to physicians and, in some cases, prescribe medication
50	Identification, understanding and prioritization of drug-drug interactions at a molecular level (e.g., use of codeine with paracetamol)
51	Identification, understanding, and prioritization of drug-patient interactions, including those that preclude or require the use of a specific drug (e.g., trastuzumab for treatment of breast cancer in women with HER2 overexpression)
52	Identification, understanding, and prioritization of drug-disease interactions (e.g., NSAIDs in heart failure)
55	Critical evaluation of the prescription to ensure that it is clinically appropriate and legal
56	Familiarity with the supply chain of medicines and the ability to ensure timely flow of drug products to the patient
58	Promotion of public health in collaboration with other actors in the healthcare system
59	Provision of appropriate lifestyle advice on smoking, obesity, *etc.*
60	Provision of appropriate advice on resistance to antibiotics and similar public health issues
61	Ability to use effective consultations to identify the patient’s need for information
62	Provision of accurate and appropriate information on prescription medicines
63	Provision of informed support for patients in selection and use of non-prescription medicines for minor ailments (e.g., cough remedies)
64	Identification and prioritization of problems in the management of medicines in a timely manner and with sufficient efficacy to ensure patient safety
66	Undertaking of a critical evaluation of prescribed medicines to confirm that current clinical guidelines are appropriately applied
67	Assessment of outcomes on the monitoring of patient care and follow-up interventions
68	Evaluation of cost effectiveness of treatment

Notes: *n* = number.

The competences ranked higher by industrial pharmacists fall into three groups with, firstly, competences 4, 6 and 18 on evaluation of scientific data, research, and production of medicines. Scores for community pharmacists on 2 of these competences (6, 18) were less than 50%. The second group was concerned with the subject matters physics (25) 33%, analytical chemistry (28) 67%, and pharmaceutical technology (34) 84%. Subject matters were included because they figure in the EU directive on the sectoral profession of pharmacy. The authors recognize that they are not competences but part of the foundation of competences. Having said that, it should be noted that community pharmacists ranked these elements very low, with a score of 22% for physics and 42% for analytical chemistry. Thus, the general message that all the subject matters cited in the EU directive are essential for science-based pharmacy practice is not understood, as concerns subjects such as physics.

In the lower part of Table 4, the competences that are ranked higher by community pharmacists are shown. Four of these concern subject areas (24, 30, 33, and 36), but the majority concern patient care competences. For the latter community pharmacists scored significantly higher than industrial pharmacists for all but five competences. This is not to say that industrial pharmacists scored low for patient care competences, as almost all of their scores were between 60% and 80%. Thus, they do recognize the need for information relating to the pharmacist as a medicine specialist (see also comments below).

Overall, the observations in the previous paragraphs suggest that the two groups are often ranking in the context of their own specific activity and, following on from this, that certain competences are needed for certain specializations. Continuing this argument further, certain competences could thus be part of an “industrial pharmacy-oriented” degree course, and others part of a “community pharmacy-oriented” degree course (taking for example, those in Table 4). An alternative argument is that this is in favor of “amplifying” different clusters in different specializations within a single curriculum, rather than “separating” competences into different curricula for the two activities.

Comments were made by 15/138 (11%) of industrial pharmacist and 23/258 (9%) of community pharmacist respondents. Our initial objective was to evaluate whether comments were in line with scoring, but the low numbers of comments received did not permit a satisfactory analysis using semi-quantitative analysis of unstructured data (results not shown). Comments are reported here, therefore, in a “raw data” form. 

Comments are grouped into areas in Table 5.

There were several comments on the English phraseology and the construction of the questions, and these have been taken into consideration in the production of the revised version of the questionnaire. Others pointed to the esoteric nature of certain competences and the recognition of specialization, be it industrial or community. Of interest also is the fact that industrial pharmacists recognized the necessity of all competences, including patient care competences, and the nature of the pharmacist as a “medicines specialist”.

**Table 5:** Comments by industrial and community pharmacists.

Area	Typical Examples of Comments Made.	
	Industrial Pharmacist.	Community Pharmacist.
Understanding of the question	- I do not understand the question. - I have not spoken English for a long time. - One point only to each question. - Important to interpret but not necessarily done.	- The question is very convoluted. - The question is rather unclear.
Production of medicines.		We buy rather than produce them.
Information sources.		- I get all my information (on drugs) from reliable sources. - Pre-selection of new scientific information by official institutes.
Framework for community pharmacy practice.	- Being a pharmacist, you need basic information in all areas. - Although I work in industry, all these competences are needed. - The pharmacist is a medicines specialist. - Response depends on the area you are working in.	- All answers refer to daily work in a community pharmacy. - Answers relate to my working environment. - Pharmaceutical care is essential. - Some competences are for specialists. - Some competences are for hospital pharmacists.
Economics/business administration.	Cost effectiveness assessment is a specialist role.	- It is vital to have economics and business administration. - “Business environment,” yes, but keep your eye on the health aspect. - Pharmacists follow recommendations of NICE not cost effectiveness.
No prescription.		- Pharmacists are not allowed to prescribe in my country. - Pharmacists are not physicians.
Healthcare team.		Pharmacists are responsible for their part of the job.
Subject areas.	- You need basic knowledge of all areas. - Pharmacognosy is no longer a required subject. - Radio-pharmacy would be useful.	We do not need analytical chemistry as we are not analyzing any more.
Industrial pharmacy/research.	- These apply to industrial pharmacists. - These competences are esoteric. - Preclinical issues are not part of my work experience.	- I have never worked in industry. - These competences are for industry and research.
Consultation/diagnosis.		- A pharmacist is not a doctor. - It is not uncommon that the pharmacist is the first person to whom the patient explains his symptoms. - We are not appropriately trained for this. - Commercial interests are involved.

Globally, the comments leave a subjective impression of backing up the scores, but, as explained in Section 2, no solid conclusions can be drawn.

## 4. Conclusions

Competences centering on values, communication skills, *etc.* were ranked similarly by the two groups of pharmacists (industrial and community). In other areas, such as (1) drug research, development and production, and (2) patient care, scores suggest that the groups appeared to be ranking in the context of their own specific activity. These results are discussed in the light of the existence of, or need for, an “industrial pharmacy” specialization based on a specific competence framework. The latter is provided by the PHAR-QA framework.

The pharmacists’ perception of their profession is primarily determined within the context of their specific activities in their line of work. The split in opinion lies between “hard sciences” and “patient care.” The truth is that the pharmacy service that best serves the population at large involves both. Pharmacy students seldom know in advance whether they will end up working as industrial or as community pharmacists. Taking this into account, one could argue that a pharmacist should receive a balanced education involving the two areas and then specialize in one of the two as his/her professional career advances, post-registration. On the other hand, if the degree of specialization necessary is so profound that it would be best to start it from the educational phase, the specialty-oriented program may be the better solution. The discussion on this dilemma continues.

## 5. Perspectives

In light of the rankings and comments, a revised version of the competence framework will be sent out for survey. This will be followed by the proposal of a PHAR-QA competence framework for pharmacy practice.

Future papers will deal with results for hospital pharmacists, and academics. These papers, as does the present paper, will deal with the implications of the results obtained, thus contributing to the ongoing debate on the perceptions of professional identity of a pharmacist [17], in this case, with contributions that are backed up by hard data.

## Appendix A. Ranking of Competences by Industrial and Community Pharmacists. (Seq.: Sequential Numbering (as in Figures).

**Table:**

Cluster	Seq.	Competence	Industrial Pharmacists	Community Pharmacists
Cluster 7. Personal competences: learning and knowledge.			83.6	79.2
	1	Ability to identify learning needs and to learn independently (including continuous professional development (CPD)).	93.3	89.8
	2	Analysis: ability to apply logic to problem solving, evaluating pros and cons and following up on the solution found.	97.0	91.1
	3	Synthesis: capacity to gather and critically appraise relevant knowledge and to summarise the key points.	92.4	87.9
	**4**	**Capacity to evaluate scientific data in line with current scientific and technological knowledge.**	**88.1**	**75.8**
	5	Ability to interpret preclinical and clinical evidence-based medical science and apply the knowledge to pharmaceutical practice.	71.5	75.9
	**6**	**Ability to design and conduct research using appropriate methodology.**	**57.6**	**40.2**
	7	Ability to maintain current knowledge of relevant legislation and codes of pharmacy practice.	84.6	91.7
Cluster 8. Personal competences: values.			91.7	93.7
	8	Demonstrate a professional approach to tasks and human relations.	93.9	94.5
	9	Demonstrate the ability to maintain confidentiality.	96.9	95.3
	10	Take full personal responsibility for patient care and other aspects of one’s practice.	86.7	94.8
	11	Inspire the confidence of others in one’s actions and advice.	86.0	88.8
	12	Demonstrate high ethical standards.	94.7	95.2
Cluster 9. Personal competences: communication and organizational skills.			78.7	76.3
	13	Effective communication skills (both orally and written).	89.3	94.8
	14	Effective use of information technology.	86.3	86.1
	15	Ability to work effectively as part of a team.	89.1	89.2
	16	Ability to identify and implement legal and professional requirements relating to employment (e.g., for pharmacy technicians) and to safety in the workplace.	82.2	81.0
	17	Ability to contribute to the learning and training of staff.	77.1	82.5
	**18**	**Ability to design and manage the development processes in the production of medicines.**	**72.8**	**43.2**
	19	Ability to identify and manage risk and quality of service issues.	78.9	79.2
	20	Ability to identify the need for new services.	62.9	64.5
	21	Ability to communicate in English and/or locally relevant languages.	85.2	74.1
	22	Ability to evaluate issues related to quality of service.	80.2	77.9
	23	Ability to negotiate, understand a business environment and develop entrepreneurship.	61.2	64.1
Cluster 10. Personal competences: knowledge of different areas of the science of medicines.			63.8	66.1
	**24**	**Plant and animal biology.**	**23.3**	**39.3**
	**25**	**Physics.**	**33.3**	**21.7**
	26	General and inorganic chemistry.	53.1	43.9
	**27**	Organic and medicinal/pharmaceutical chemistry.	76.7	66.0
	**28**	**Analytical chemistry.**	**67.2**	**41.9**
	29	General and applied biochemistry (medicinal and clinical).	71.9	68.8
	**30**	**Anatomy and physiology; medical terminology.**	**70.3**	**88.7**
	**31**	Microbiology.	59.2	72.2
	**32**	Pharmacology including pharmacokinetics.	88.4	94.7
	**33**	**Pharmacotherapy and pharmaco-epidemiology.**	**75.8**	**94.3**
	**34**	**Pharmaceutical technology including analyses of medicinal products.**	**84.4**	**62.0**
	35	Toxicology.	65.1	74.0
	**36**	**Pharmacognosy.**	**37.5**	**66.5**
	37	Legislation and professional ethics.	86.8	89.5
Cluster 11. Personal competences: understanding of industrial pharmacy.			70.7	59.7
	**38**	**Current knowledge of design, synthesis, isolation, characterization and biological evaluation of active substances.**	**57.1**	**41.7**
	**39**	**Current knowledge of good manufacturing practice (GMP) and of good laboratory practice (GLP).**	**81.9**	**59.4**
	**40**	**Current knowledge of European directives on qualified persons (QPs).**	**66.7**	**43.7**
	**41**	**Current knowledge of drug registration, licensing and marketing.**	**84.1**	**55.7**
	42	Current knowledge of good clinical practice (GCP).	63.5	64.5
Cluster 12. Patient care competences: patient consultation and assessment.			52.0	77.0
	**43**	**Ability to perform and interpret medical laboratory tests.**	**44.4**	**65.5**
	**44**	**Ability to perform appropriate diagnostic or physiological tests to inform clinical decision making e.g., measurement of blood pressure.**	**40.9**	**73.6**
	**45**	**Ability to recognise when referral to another member of the healthcare team is needed because a potential clinical problem is identified (pharmaceutical, medical, psychological or social).**	**71.9**	**91.7**
Cluster 13. Patient care competences: need for drug treatment.			66.0	87.0
	**46**	**Retrieval and interpretation of relevant information on the patient’s clinical background.**	**63.3**	**84.0**
	**47**	**Retrieval and interpretation of an accurate and comprehensive drug history if and when required.**	**69.6**	**91.5**
	**48**	**Identification of non-adherence and implementation of appropriate patient intervention.**	**63.8**	**86.8**
	**49**	**Ability to advise to physicians and—in some cases—prescribe medication.**	**66.7**	**87.6**
Cluster 14. Patient care competences: drug interactions.			69	93
	**50**	**Identification, understanding and prioritization of drug-drug interactions at a molecular level (e.g., use of codeine with paracetamol).**	**70.4**	**91.6**
	**51**	**Identification, understanding, and prioritization of drug-patient interactions, including those that preclude or require the use of a specific drug (e.g., trastuzumab for treatment of breast cancer in women with HER2 overexpression).**	**64.9**	**89.7**
	**52**	**Identification, understanding, and prioritization of drug-disease interactions (e.g., NSAIDs in heart failure).**	**71.7**	**96.6**
Cluster 15. Patient care competences: provision of drug product.			73.1	83.3
	53	Familiarity with the bio-pharmaceutical, pharmacodynamic and pharmacokinetic activity of a substance in the body.	70.4	81.2
	54	Supply of appropriate medicines taking into account dose, correct formulation, concentration, administration route and timing.	85.2	94.9
	**55**	**Critical evaluation of the prescription to ensure that it is clinically appropriate and legal.**	**77.6**	**94.0**
	**56**	**Familiarity with the supply chain of medicines and the ability to ensure timely flow of drug products to the patient.**	**71.1**	**84.6**
	57	Ability to manufacture medicinal products that are not commercially available.	60.6	60.5
Cluster 16. Patient care competences: patient education.			64.0	85.0
	**58**	**Promotion of public health in collaboration with other actors in the healthcare system.**	**62.8**	**82.6**
	**59**	**Provision of appropriate lifestyle advice on smoking, obesity, *etc.***	**57.1**	**80.9**
	**60**	**Provision of appropriate advice on resistance to antibiotics and similar public health issues.**	**73.0**	**93.1**
Cluster 17. Patient care competences: provision of information and service.			73.0	93.0
	**61**	**Ability to use effective consultations to identify the patient’s need for information.**	**68.5**	**90.9**
	**62**	**Provision of accurate and appropriate information on prescription medicines.**	**80.9**	**94.4**
	**63**	**Provision of informed support for patients in selection and use of non-prescription medicines for minor ailments (e.g., cough remedies...).**	**70.9**	**94.0**
Cluster 18. Patient care competences: monitoring of drug therapy.			74.0	86.0
	**64**	**Identification and prioritization of problems in the management of medicines in a timely manner and with sufficient efficacy to ensure patient safety.**	**76.9**	**93.0**
	65	Ability to monitor and report to all concerned in a timely manner, and in accordance with current regulatory guidelines on Good Pharmacovigilance Practices (GVPs), Adverse Drug Events and Reactions (ADEs and ADRs).	81.1	83.4
	**66**	**Undertaking of a critical evaluation of prescribed medicines to confirm that current clinical guidelines are appropriately applied.**	**65.1**	**80.6**
Cluster 19. Patient care competences: evaluation of outcomes.			54.0	70.0
	**67**	**Assessment of outcomes on the monitoring of patient care and follow-up interventions.**	**60.2**	**79.0**
	**68**	**Evaluation of cost effectiveness of treatment.**	**47.3**	**61.2**

Notes: Competences in bold are those showing a statistically significant difference in distribution of rankings between groups (chi-square, *p* < 0.05).
